# Endocannabinoid System and the Kidneys: From Renal Physiology to Injury and Disease

**DOI:** 10.1089/can.2018.0060

**Published:** 2019-03-13

**Authors:** Janice T. Chua, Donovan A. Argueta, Nicholas V. DiPatrizio, Csaba P. Kovesdy, Nosratola D. Vaziri, Kamyar Kalantar-Zadeh, Hamid Moradi

**Affiliations:** ^1^ University of California–Irvine, School of Medicine, Orange, California.; ^2^ Division of Biomedical Sciences, School of Medicine, University of California, Riverside, Riverside, California.; ^3^ Division of Nephrology, University of Tennessee Health Science Center, Memphis, Tennessee.; ^4^ Nephrology Section, Memphis Veterans Affairs Medical Center, Memphis, Tennessee.; ^5^ Nephrology Section, Tibor Rubin Veteran Affairs Health System, Long Beach, California.

**Keywords:** acute kidney injury, chronic kidney disease, endocannabinoid, fibrosis, inflammation, nephropathy

## Abstract

**Introduction:** As the prevalence of kidney disease continues to rise worldwide, there is accumulating evidence that kidney injury and dysfunction, whether acute or chronic, is associated with major adverse outcomes, including mortality. Meanwhile, effective therapeutic options in the treatment of acute kidney injury (AKI) and chronic kidney disease (CKD) have been sparse. Many of the effective treatments that are routinely utilized for different pathologies in patients without kidney disease have failed to demonstrate efficacy in those with renal dysfunction. Hence, there is an urgent need for discovery of novel pathways that can be targeted for innovative and effective clinical therapies in renal disease states.

**Discussion:** There is now accumulating evidence that the endocannabinoid (EC) system plays a prominent role in normal renal homeostasis and function. In addition, numerous recent studies have described mechanisms through which alteration in the EC system can contribute to kidney damage and disease. These include a potential role for cannabinoid receptors in tubulo-glomerular damage and fibrosis, which are common features of AKI, interstitial nephritis, glomerulopathy, and other conditions leading to AKI and CKD.

**Conclusion:** These findings suggest that manipulating the EC system may be an effective therapeutic strategy for the treatment of kidney disease and injury. However, further mechanistic studies are needed to fully delineate the role of this system in various conditions affecting the kidneys. Furthermore, while most of the current literature is focused on the role of the EC system as a whole in renal pathophysiology, future studies will also need to clarify the contribution of each component of this system, including the EC mediators, in the pathogenesis of kidney disease and their potential role as part of a therapeutic strategy.

## Introduction

The kidneys play a central role in normal body homeostasis through a variety of functions, including removal of byproducts of metabolism, clearance of toxins, regulation of body volume status, electrolytes and systemic hemodynamics, and production of hormones such as erythropoietin and active vitamin D. Hence, it is not surprising that kidney damage is associated with significant morbidity and mortality. The latter is true whether the decline in renal function is part of an acute process such as acute kidney injury (AKI) due to tubular necrosis, or a more chronic process such as chronic kidney disease (CKD) caused by hypertension (HTN) or diabetes. Furthermore, the mechanisms responsible for renal injury are complex and can be varied. While these mechanisms are regularly categorized based on the type of injury (acute or chronic) and anatomic part of the nephron affected (including the glomerulus, tubules, mesangium, vasculature), there is significant overlap between these categories. For instance, there is evidence indicating that AKI can result in CKD. In addition, there is frequent overlap between the different anatomic sites of injury given that damage to one part of the nephron over a period of time can result in injury to other sites. For example, while diabetic kidney disease often manifests with glomerular injury and proteinuria, over an extended period of time it also results in tubulointerstitial damage and fibrosis leading to progressive CKD and end-stage kidney disease. Therefore, understanding the underlying pathways whose alterations can result in various forms of renal damage and injury can play an important role in devising effective therapies to prevent and treat kidney disease. In this regard, there is accumulating evidence that indicates that the endocannabinoid (EC) system plays a major role in normal renal physiology. In addition, there are data demonstrating that alterations of this pathway can lead to the pathogenesis of both acute and chronic kidney disease. Therefore, evaluation of the EC system can be a promising area of discovery, which may result in the generation of potentially novel therapies aimed at treating various forms of kidney disease.

The EC system comprises endogenous fatty acid-derived ligands, their receptors, and the enzymes required for their biosynthesis and degradation.^1^ The most well-characterized ECs are *N*-arachidonoyl ethanolamide, also known as anandamide (AEA), and 2-arachidonoyl-*sn*-glycerol (2-AG).^2^ These lipid-derived molecules are generated on-demand by the metabolism of membrane phospholipids in response to various stimuli, including elevated intracellular calcium or metabotropic receptor activation.^3^ After production, they bind to the local cannabinoid receptors in an autocrine or paracrine manner, although measurable concentrations of these ligands can also be found in the blood, cerebrospinal fluid, and lymph.^4^ While the potential endocrine actions of these ECs remain an area of active research, it is well established that they act locally by binding with two widely studied cannabinoid receptors, cannabinoid subtype-1 (CB_1_) and subtype-2 (CB_2_).^5^ AEA and 2-AG can subsequently be taken up by cells through a high-affinity uptake mechanism^6,7^ and rapidly degraded through the action of the enzymes, fatty acid amide hydrolase (FAAH), and monoacylglycerol lipase (MGL), respectively.^1^

While the role of the EC system has been initially a focus of extensive research in the central nervous system, over the course of the past two decades, a significant number of studies have confirmed its presence and importance in the peripheral organs, including the kidneys. In this regard, substantial concentrations of ECs, the machinery required for their biosynthesis and degradation, as well as CB receptors have been detected in kidney tissue.^8,9^ The effects produced by the actions of this system in the normal and pathological conditions of the kidney, however, have not been fully delineated given the many complexities involved in the production and breakdown of EC ligands.^8–11^ In addition, the differential distribution and actions of the CB_1_ and CB_2_ receptors in various structures and cell subtypes in the kidney can ultimately result in varied signaling outcomes whose overall impact will be difficult to predict. Accordingly, identifying the physiologic and pathophysiologic roles of the EC system in the field of nephrology remains an active area of exploration.

## EC System and Normal Renal Physiology

It has been shown that CB_1_ and CB_2_ belong to a class of seven transmembrane domain G-protein-coupled receptors that are functionally dependent on the activation of heterotrimeric G_i_/G_0_ proteins.^11^ Although the activation of both receptors results in the inhibition of adenylyl cyclase enzyme and increased activity of mitogen-activated protein kinase (MAPK), CB_1_ activation has also been shown to stimulate nitric oxide synthase and directly control the activation of ion channels. The latter include the inwardly rectifying and A-type outward potassium channels, D-type outward potassium channels, and N-type and P/Q-type calcium channels.^10,12,13^ Despite the common G-protein subunit shared between CB_1_ and CB_2_ receptors, their activation can produce opposing biological effects in normal and diseased states, in part due to the abundance and localization of these cannabinoid receptors and their EC ligands.

**FIG. 1.**
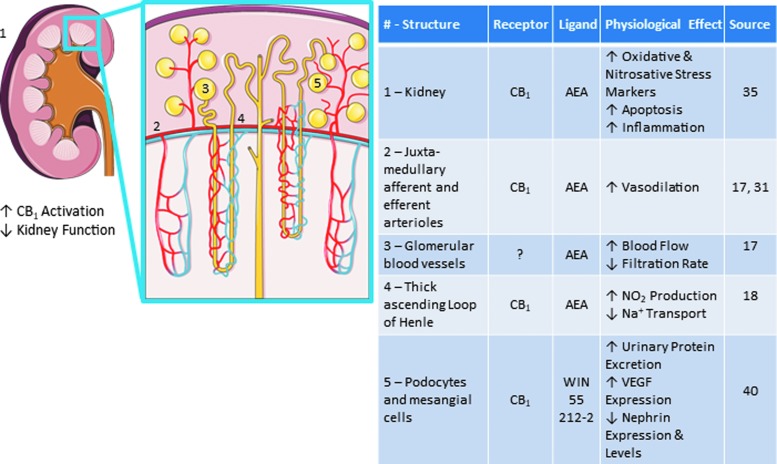
Roles of the endocannabinoid system in healthy kidney. Images adapted from Servier Medical Art “Kidney” licensed under CC 3.0, https://smart.servier.com/smart_image/kidney

While the CB_1_ receptor was initially thought to be localized to the central and peripheral nervous system,^12,14^ it has been shown to be present in peripheral organs such as the kidneys.^15,16^ For instance, the presence of functional CB_1_ receptor has been demonstrated in proximal convoluted tubules, distal tubules, and intercalated cells of the collecting duct in the human kidney^13^ (Fig. 1). Furthermore, CB_1_ receptor expression has also been found in other parts of the nephron in rodents, such as the afferent and efferent arterioles,^17^ thick ascending limbs (TAL) of the loop of Henle,^18^ and glomeruli,^19–23^ as well as in various kidney cell subtypes such as glomerular podocytes,^24,25^ tubular epithelial cells,^13,15,20,21,24,26–29^ and cultured mesangial cells.^30,31^ Similarly, the expression of CB_2_ receptors, although previously thought to be predominantly in immune cells,^32^ has also been demonstrated in renal tissue.^33^ For example, CB_2_ receptor expression has been localized to podocytes,^25^ proximal tubule cells,^26,33,34^ and mesangial cells^31^ in human and rat renal cortex samples.

In addition to differential expression of CB receptors in different tissues and cells, the complex regulation of the biosynthesis and degradation of the kidney's high basal levels of ECs through downstream enzymes contributes to the varied signaling effects of these ligands.^20,31,35–39^ While the renal cortex displayed similar levels of AEA and 2-AG, AEA was demonstrated to be enriched in the kidney medulla compared with the cortex, while the levels of 2-AG in the medulla were similar to those of both ECs in the cortex.^39^ Moreover, AEA is present in cultured renal endothelial and mesangial cells at low levels and can be synthesized from arachidonic acid and ethanolamine and catabolized by AEA amidase in these kidney cell subtypes.^31^ The expression of FAAH was shown to be augmented in the renal cortex (e.g., in the glomerulus, tubular system, and collecting ducts) in comparison to its low expression levels in the medulla.^39^

Considering the diverse localization of the ECs and their receptors, as well as the complexities involved in their synthesis and catabolism, this system can play various roles in kidney function. Under normal conditions, the EC system is capable of regulating renal homeostasis as demonstrated by its control over renal hemodynamics, tubular sodium reabsorption, and urinary protein excretion. These effects are largely imparted through the activation of the CB_1_ receptor.^17,18,31,39–41^ In the following sections, we describe some effects of EC system activation on renal physiologic function (Fig. 1).

### Renal hemodynamics

Under normal physiologic conditions, the EC system plays a critical role in the regulation of renal hemodynamics. For instance, it was shown that intravenous administration of AEA decreased glomerular filtration rate and increased renal blood flow in rodents, independent of changes in blood pressure.^17^ *In vitro* studies showed that AEA can vasodilate juxtamedullary afferent or efferent arterioles^17,31^ through a CB_1_-dependent process, normally inhibited by nitric oxide synthase,^31^ to regulate glomerular filteration rate (GFR). The actions of the AEA signaling system are likely conducted through endothelial and mesangial cells, which are capable of producing and metabolizing AEA,^31^ as well as through the hyperpolarization of smooth muscle cells via the activation of potassium channels.^42^ It should be noted that there are also non-CB_1_ receptor–dependent mechanisms by which ECs can mediate a vasodilatory effect and thereby regulate renal hemodynamics.^43^ Future studies need to further elucidate the role of the latter mechanisms in normal renal physiologic homeostasis.

### Tubular sodium transport

AEA has been shown to have a regulatory effect on tubular sodium transport. In the medullary TAL of Henle's loop, AEA (through interaction with the CB_1_ receptor) was shown to stimulate nitric oxide production, leading to an inhibition of sodium transport through the apical Na^+^/H^+^ transporter and Na^+^/K^+^/2Cl^−^ co-transporter. This was also associated with reduced oxygen consumption in the TAL portion of the nephron.^18^ This suggests that the activation of CB receptors via AEA can regulate renal blood flow as well as tubular handling of solutes, which can ultimately impact renal salt and water clearance.

### Urinary protein excretion and modulation

To examine the role of glomerular CB_1_ receptors in modulating urinary protein excretion, Hsu et al. used CB_1_ transgenic mice and rats treated with a selective CB_1_ agonist.^40^ CB_1_ receptor activation in the kidney, and specifically in the podocytes and mesangial cells of the glomerulus, increased urinary protein excretion.^40^ Increased activation and overexpression of CB_1_ was also found to enhance vascular endothelial growth factor (VEGF) expression levels and subsequently reduce nephrin gene and protein levels, suggesting a potential pathway for podocyte dysregulation and proteinuria.^40^

## EC System and Renal Disease

**FIG. 2.**
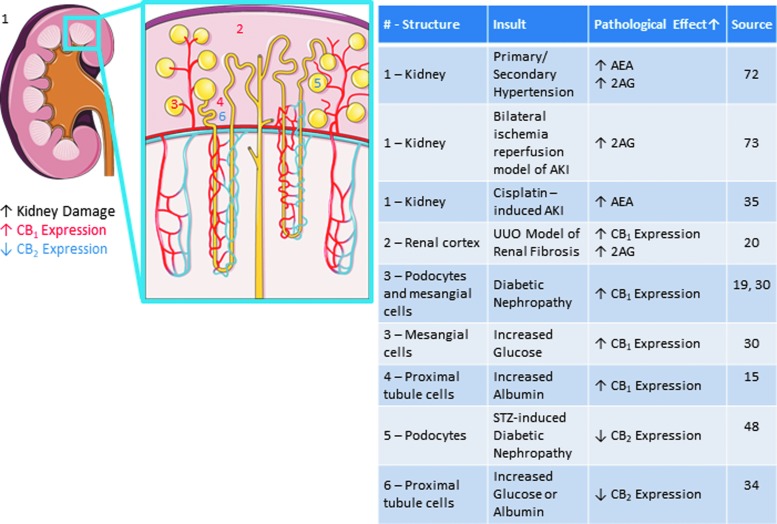
Impact of various kidney disease models on receptors and ligands of the endocannabinoid system. Images adapted from Servier Medical Art “Kidney” licensed under CC 3.0, https://smart.servier.com/smart_image/kidney

The role of the EC system in renal pathology and dysfunction is an emerging area of research, which has been studied primarily in the context of CB receptors. Alterations of CB receptor expression and activity have been discovered in various renal diseases such as diabetic nephropathy, CKD, and different types of kidney injury (Fig. 2). Collectively, these studies on renal pathophysiology suggest that targeting the EC system may be of diagnostic and therapeutic value (Tables 1 and 2).

**Table 1.** Impact of Targeting Cannabinoid Receptors in the Models of Kidney Disease

Mediator	Treatment	Location	Effect	Disease model	Source
CB_1_	AM251 (inverse agonist)		↓ Albuminuria	Mouse STZ-induced diabetic nephropathy	^19^
CB_1_	Rimonabant (inverse agonist)	Glomeruli and proximal tubules	↓ Apoptosis	Rat STZ-induced diabetic nephropathy	^19,21,29^
CB_2_	AM1421 (agonist)	Podocytes	↓ Albuminuria ↓ Monocyte infiltration ↓ Expression of profibrotic markers	Mouse/rat obesity-related nephropathy	^25,33^
CB_2_	HU910 (agonist)	Podocytes	–Restored nephrin expression ↓ Albuminuria ↓ Mesangial matrix formation ↓ Fibronectin accumulation ↓ Sclerotic damage	ob/ob mice	^49^
CB_1_	JD5037 (inverse agonist)		↑ Renal structure and function ↓ Proteinuria	ZDF rats	^24^
CB_1_	AM251 rimonabant (inverse agonist)		↓ Body weight ↓ Systolic blood pressure ↓ Circulating leptin ↓ Albuminuria ↓ Circulating creatinine	DIO rats db/db mice	^16,23^
CB_1_	Rimonabant (inverse agonist)		↓ Glomerular fibrosis ↓ Tubular damage ↓ Renal hypertrophy	DIO rats	^21,52–54^
CB_1_	Knockout	Renal proximal tubule cells	↓ Lipid accumulation ↓ Liver kinase B1 activation ↓ AMP-activated protein kinase activity ↓ Fatty acid beta-oxidation	DIO mice	^55^
CB_1_	Rimonabant (inverse agonist)		↓ Monocyte chemoattractant protein-1 ↓ Macrophage infiltration	Mouse UUO	^20^
CB_1_	Win55,212-2 (agonist)	Glomeruli	↑ VEGF	Mouse/rat UUO	^40^
CB_1_/CB_2_	ACPA/JWH133 (agonists)	Tubules	–Prevented damage	Mouse renal ischemia/reperfusion injury	^56^
CB_1_/CB_2_	AM281/BCP (antagonist/agonist)		↓ Oxidative stress ↓ Tubular damage	Mouse cisplatin-induced renal damage	^35,59–61^
CB2	LEI-101 (agonist)		↓ Serum BUN ↓ Serum creatinine	Cisplatin-induced renal damage	^59^
CB2	Knockout		↑ Mortality ↑ Lung injury ↑ Bacteremia ↑ Neutrophil recruitment ↓ p38 MAPK at injury site	CLP model of sepsis in mouse	^64^
CB_1_?	Palmitic acid	Proximal tubule cells	↓ Apoptosis	Human hyperlipidemia/diabetic nephropathy	^29^
?	CBD	Tubules	↓ Serum creatinine ↓ Renal malondialdehyde ↓ Nitric oxide	Rat renal ischemia/reperfusion injury	^57^

Arrows represent an observed change *p*<0.05. Question mark represents unconfirmed or unknown mediator.

BUN, blood urea nitrogen; CBD, cannabidiol; CLP, cecal ligation and puncture; STZ, streptozotocin; UUO, unilateral ureteral obstruction; VEGF, vascular endothelial growth factor; ZDF, Zucker diabetic fatty.

**Table 2.** Impact of Endocannabinoid Manipulation in the Models of Kidney Disease

Ligand	Treatment	Location	Effect	Disease model	Source
AEA/2AG	URB597 (FAAH inhibitor)	Whole kidney	–Inhibited ROS generation –Augmented ROS-dependent phospholipid peroxidation	Rat primary/secondary HTN	^72^
AEA/2AG	URB597 (FAAH inhibitor)	Whole kidney	↑ Antioxidant defense ↑ CB_1_/CB_2_ expression	Rat primary HTN	^72^
AEA/2AG	URB597 (FAAH inhibitor)	Whole kidney	↓ Pro-inflammatory responses ↑ CB_2_ expression ↑ TRPV1 expression	Rat secondary HTN	^72^
2AG	JZL184 (MGL inhibitor)	Serum Medulla	↓ Serum BUN ↓ Serum creatinine ↑ Tubular damage score	Mouse ischemia/reperfusion model of AKI	^73^
AEA/2AG	Administration	Human cultured podocytes	↑ Inflammation and injury ↓ Podocin ↓ Nephrin ↑ Desmin gene expression	Chronic high-glucose exposure	^74^
AEA	Administration	Mouse cultured podocytes	–Blocked NLRP3 inflammasome activation –Ameliorated podocyte dysfunction	l-Hcys-induced podocyte injury	^75^

2-AG, 2-arachidonoyl-*sn*-glycerol; AEA, anandamide; AKI, acute kidney injury; CB_1_, cannabinoid subtype-1; CB_2_, cannabinoid subtype-2; FAAH, fatty acid amide hydrolase; HTN, hypertension; Hcys, l-homocysteine; MGL, monoacylglycerol lipase; ROS, reactive oxygen species.

### Diabetic nephropathy

It is well known that diabetes has major renal complications, including progressive kidney disease and pathology, a condition known as diabetic nephropathy. Diabetic nephropathy is characterized by glomerular hypertrophy and hyperfiltration, which can result in albuminuria, renal fibrosis, GFR decline, and end-stage renal disease.^44,45^ Several studies have examined the role of the EC system in diabetes-related podocyte, mesangial and tubular cell injury, as well as the function of CB receptor activation on the adverse outcomes of diabetic nephropathy (Fig. 2).

The evaluation of mouse models of diabetic kidney disease and renal tissue from humans with advanced diabetic nephropathy have shown elevated levels of CB_1_ receptor expression in the kidney, and in particular in glomerular podocytes and mesangial cells.^19,30^ In addition, *in vitro* studies have shown CB_1_ receptor upregulation with exposure to increased glucose and albumin concentrations in mesangial cells^30^ and proximal tubule cells, respectively.^15^ Furthermore, the CB_1_ receptor has been found to be overexpressed in glomerular podocytes in experimental mice with diabetic nephropathy.^19,24^ The potential consequences of the latter changes were shown in another study that found that hyperlipidemia, as induced by diabetic nephropathy, can be associated with palmitic acid–induced apoptosis in proximal tubular cells. These actions are mediated through upregulated CB_1_ receptor expression.^29^

Given the evidence indicating a deleterious role for the CB_1_ receptor in diabetic nephropathy, several studies have investigated the utility of CB_1_ antagonist/inverse agonists as a potential therapeutic option for diabetic kidney disease.^15,20,24,46,47^

In a streptozotocin (STZ)-induced mouse model of diabetic nephropathy, albuminuria was reduced as a result of CB_1_ receptor blockade through a selective CB_1_ receptor antagonist.^19^ Similar findings were also reported in genetic mouse models of diabetic nephropathy.^23,24^ It was found that a marked reduction in proteinuria occurred through the preservation of glomerular podocytes and restoration of the expression of podocyte proteins nephrin, podocin, and zonula occludens-1.^19,24^ In addition, CB_1_ antagonism was also found to be associated with decreased glomerular and proximal tubular apoptosis, ultimately leading to improvements in renal function.^19,21,29^

In Zucker diabetic fatty (ZDF) rats, which develop type 2 diabetes due to obesity caused by a dysfunctional leptin receptor, chronic administration of a CB_1_ receptor inverse agonist restored GFR, reduced proteinuria, and improved the markers of podocyte health through modulation of the renin–angiotensin system and inhibition of apoptosis.^24^

While diabetic kidney disease is associated with increased expression of the CB_1_ receptor in various parts of the nephron, there is also evidence that CB_2_ receptor expression is significantly reduced. For example, STZ-induced diabetic nephropathy in mice is associated with the downregulation of glomerular podocyte CB_2_ receptor expression.^48^ Similarly, there is decreased expression of the CB_2_ receptor in proximal tubule cells following exposure to elevated concentrations of albumin and glucose.^34^ Furthermore, CB_2_ receptor activation has been shown to ameliorate albuminuria, restore podocyte protein expression, reduce monocyte infiltration, and decrease the expression of renal profibrotic markers^25,33^ in rats with obesity-related nephropathy. CB_2_ agonism in obese diabetic nephropathy BTBR ob/ob mouse strain also reduced albuminuria, ameliorated dysfunctional nephrin expression in podocytes, and reduced mesangial matrix expansion, fibronectin accumulation, and sclerotic damage.^49^

These studies demonstrate that antagonism of CB_1_ receptors and activation of CB_2_ receptors using selective pharmacological ligands is associated with the restoration of renal structure and function, specifically albuminuria and the expression of inflammatory markers, in genetic and experimental models of diabetic nephropathy.

### Obesity-related kidney disease

Obesity is associated with and acts as a risk factor for the development of diabetic nephropathy,^50^ with obese individuals possessing a higher risk of progressing to end-stage renal disease.^51^ A study by Jenkin et al.^33^ revealed a role of CB_2_ receptor activation in reducing the progression of obesity-related kidney dysfunction by decreasing proteinuria, creatinine clearance, and renal fibrotic markers. In contrast, ZDF rats treated with a CB_1_ inverse agonist showed improved renal structure and function.^24^ Surprisingly, these rats also displayed a marked increase in body weight compared with the weight stability of control mice, which was thought to be due to the development of extreme hyperglycemia in vehicle-treated controls.^24^ In a separate study, CB_1_ antagonism was shown to decrease albuminuria, reduce mesangial expansion, and ameliorate the expression of profibrotic and proinflammatory kidney proteins in lean and obese diabetic mouse models.^23^ These findings suggest that the consequences of CB_1_ modulation in diabetes can differ based on the experimental model, presence of obesity, and presence of hyperglycemia.

In other studies that utilized rats with diet-induced obesity, CB_1_ receptor expression in the kidney is notably upregulated, and treatment with a CB_1_ receptor antagonist reduced weight, systolic blood pressure, plasma leptin, albuminuria, and plasma creatinine levels. This is associated with the amelioration of glomerulopathy.^16^ Furthermore, studies using obese Zucker rats demonstrated that the CB_1_ inverse agonist, rimonabant, ameliorated proteinuria in an animal model of obesity-induced nephropathy.^21^ Treatment with rimonabant partially restored creatinine clearance, reduced glomerulosclerosis and tubular-interstitial fibrosis, and lowered tubular damage and renal hypertrophy.^21^ It should also be noted that these findings may have been mediated by the effects of rimonabant and not related to the EC system. While obesity in fa/fa Zucker rats is caused by a mutation of the leptin receptor, rimonabant acts to increase leptin uptake by the kidney, which has been shown to reduce proximal tubule metabolic activity.^52^ Therefore, improvement in renal function in these rats may have occurred due to mechanisms related to leptin's role in proximal tubule cell metabolism,^52–54^ as opposed to a direct action on the EC system.

Using a novel mouse strain lacking CB_1_ receptors in renal proximal tubule cells, Udi et al.^55^ found that CB_1_ receptor deletion did not protect the mice from the deleterious metabolic effects associated with obesity, but significantly diminished obesity-induced lipid accumulation in the kidney. Furthermore, the stimulation of CB_1_ receptors in renal proximal tubule cells was found to be associated with decreased activation of liver kinase B1 and decreased activity of AMP-activated protein kinase, as well as reduced fatty acid beta-oxidation.^55^ These findings indicate a potential relationship between renal proximal tubular epithelial cell CB_1_ receptor and the pathologic effects of obesity-induced renal lipotoxicity and nephropathy.

In summary, the findings related to the CB_1_ receptor highlight its partial potential in acting as a therapeutic target for obesity-induced renal disease. Further studies are needed to ascertain the efficacy of modulating CB_1_ in the kidney to improve renal dysfunction independent of its effects on weight.

### Renal interstitial disease and fibrosis

The CB_1_ receptor has been shown to be upregulated in other renal disorders marked by interstitial inflammation and fibrosis, including acute interstitial nephritis.^20^ Using unilateral ureteral obstruction (UUO) as an experimental model for renal fibrosis in mice, Lecru et al.^20^ showed that CB_1_ receptor expression was upregulated in UUO animals compared with controls. This is also associated with a marked increase in the renal content of 2-AG. Treatment of UUO mice with rimonabant reduced monocyte chemoattractant protein-1 synthesis and decreased macrophage infiltration.^20^ It was also shown that CB_1_ receptor activation led to enhanced VEGF levels, which subsequently reduced nephrin expression and protein levels.^40^

### Acute kidney injury

There is accumulating evidence indicating the important role of CB_1_ and CB_2_ receptors and their modulation in the pathogenesis of various forms of AKI. With regard to ischemic AKI, selective CB_1_ and CB_2_ receptor agonists were found to have a dose-dependent effect in preventing tubular damage following renal ischemia/reperfusion injury in mouse kidney.^56^ In a separate study, however, the administration of cannabidiol, a non-psychoactive constituent of cannabis with poorly defined pharmacological properties, led to a reduction in renal tubular injury in rats following bilateral renal ischemia/reperfusion.^57^ Cannabidiol significantly attenuated the elevation of serum creatinine and renal malondialdehyde and nitric oxide levels associated with this condition.^57^ In a more recent study, a triazolopyrimidine-derived CB_2_ receptor agonist was demonstrated to play a protective role in inflammatory renal injury following bilateral kidney ischemia/reperfusion.^58^

A series of studies have demonstrated the deleterious role of CB_1_ and the protective effects of CB_2_ activation on a nephrotoxic model of AKI in cisplatin-induced renal injury.^35,59–61^ Inhibiting CB_1_ receptor^35^ or activating CB_2_ receptor^59,60^ limited oxidative stress and inflammation and reduced tubular damage in kidneys of animals with cisplatin-induced AKI. In addition, ß-Caryophyllene, a natural agonist of CB_2_ receptor, dose-dependently protected against the deleterious effects of cisplatin-induced nephrotoxicity.^61^

Furthermore, CB_1_ and CB_2_ receptors have been shown to play a role in renal apoptotic and inflammatory signaling pathways.^35,59,60^ Indeed, activating CB_1_ receptors is known to result in enhanced expression of oxidative/nitrosative stress markers, which activate p38, MAPK, and c-Jun N-terminal kinase pathways, as well as nuclear factor kappa-light-chain-enhancer of activated B cells-dependent transcription of downstream proinflammatory target genes. Ultimately, the activation of either route leads to apoptotic cell death and inflammation in the kidney.^35^ Conversely, CB_2_ receptor activation has been found to reduce proapoptotic signaling^59,60^ and mediate anti-inflammatory effects by attenuating immune cell infiltrates and inflammatory cytokine release.^59^ Mukhopadhyay et al.^59^ showed that a peripherally restricted CB_2_ receptor agonist (LEI-101), in a mouse model of cisplatin-induced nephrotoxicity, dose-dependently attenuated renal dysfunction as measured by serum concentrations of blood urea nitrogen (BUN) and creatinine. The protective effects of CB_2_ receptor activation in these studies were absent in CB_2_ receptor knockout mice, suggesting that CB_2_ receptors are a promising therapeutic target for reducing renal inflammation, oxidative/nitrosative stress, and apoptosis.

Another major contributor to AKI, which is associated with significant morbidity and mortality, is sepsis-associated kidney injury (SA-AKI).^62,63^ In a study using a cecal ligation and puncture (CLP) mouse model of sepsis, CB_2_ receptor knockout mice demonstrated increased mortality, lung injury, bacteremia, neutrophil recruitment, and decreased p38 MAPK activity at the site of infection.^64^ Treatment with a selective CB_2_ receptor agonist reduced the effects caused by CLP, such as inflammation, lung damage, and neutrophil recruitment, and ultimately improved survival.^64^ These findings are in line with evidence demonstrating that following CB_2_ localization to leukocytes, their activation has been shown to mitigate leukocyte tumor necrosis factor-α-induced endothelial cell activation, adhesion and migration of leukocytes, as well as proinflammatory modulators.^65–68^ Therefore, CB_2_ receptor modulation may represent a novel therapeutic target in the treatment of SA-AKI.^5,69,70^

The mechanism(s) by which the cannabinoid receptors modulate or recover tubular cell survival following acute damage are not well defined at this time. However, molecular differences in cannabinoid receptor mRNA and protein levels^20,35,71^ as well as differences in the physiological outcome of receptor activation are likely related to the type of AKI and to the abundance and localization of receptors.

#### EC ligands in renal health and disease

While many of the studies evaluating the role of the EC system in renal homeostasis and pathophysiology focused on CB receptors and their modulation, it is important to keep in mind that the overall effects of activation and inhibition of the EC system are dependent on various factors, only a portion of which is related to the activity of CB receptors. For example, the chief endogenous activators of the CB receptors, AEA and 2-AG, are present in substantial concentrations in the kidney^8,9^; however, physiological responses elicited by these ligands under normal or pathological conditions have not been fully elucidated. Furthermore, detailed studies on how elevated or decreased levels of these ligands may impact renal function and pathology are scarce. For example, it is well known that AEA plays a role in the modulation of renal hemodynamics.^17,31^ Infusion of this ligand was found to be associated with vasorelaxation of juxtamedullary afferent arterioles *in vitro*,^31^ increased renal blood flow in rodents,^17^ and alteration of tubular sodium transport.^18^ While these effects may be partly mediated through the activation of CB_1_ and CB_2_ receptors, it is important to highlight that these findings indicate the total effect of this ligand and it is difficult to identify exactly which receptors are activated in each segment of the nephron. Furthermore, there are CB receptor–independent effects that are not accounted for when the role of these ligands were to be assessed only in the context of CB receptors.

Recent studies have begun to address this important point by attempting to define the impact of these ligands in renal disease states. Biernacki et al.^72^ described alterations to the EC system in primary and secondary HTN, noting that these conditions resulted in renal oxidative stress through increased reactive oxygen species (ROS) and diminished levels of antioxidant enzymes. Despite the enhanced activity of FAAH and MGL in primary and secondary hypertensive rats, the levels of AEA and 2-AG in the kidney were significantly increased.^72^ Increasing endogenous levels of AEA by pharmacologically inhibiting its degradative enzyme, FAAH, with a selective FAAH inhibitor, URB597, was found to have resulted in the inhibition of ROS generation in both types of hypertensive rats. These effects were mediated through improvement in antioxidant defense in the primary spontaneously hypertensive rat (SHR) kidney via the Nrf2 pathway, as well as through reduced proinflammatory responses in secondary hypertensive (DOCA-salt) rats.^72^ Furthermore, URB597 augmented ROS-dependent phospholipid peroxidation products and levels of ECs in both types of hypertensive kidneys, which resulted in enhanced CB receptor expression in SHR rats and enhanced expression of CB_2_ and TRPV1 receptors in DOCA-salt rats.^72^ Chronic treatment of Wistar normotensive control rats with URB597 similarly enhanced phospholipid oxidation in the kidney, comparable to its administration in DOCA-salt rats.^72^ Thus, while the EC system appears to play a protective role in HTN, the administration of a FAAH inhibitor did not significantly alter the proinflammatory or oxidative conditions caused by primary HTN, and only created imbalances between ECs, oxidants, and proinflammatory factors in secondary HTN, potentially leading to the development of kidney dysfunction.

With regard to other renal conditions, such as AKI, studies have shown varied responses to kidney injury in EC expression levels. Moradi et al.^73^ demonstrated that renal ischemia/reperfusion injury is associated with a significant increase in renal 2-AG content using a bilateral ischemia/reperfusion mouse model of AKI. It was found that the augmentation of kidney 2-AG concentrations following MGL inhibitor administration resulted in improved serum BUN, creatinine, and tubular damage score; however, the mRNA gene expression of renal inflammation and oxidative stress markers was not altered. Conversely, in a cisplatin-induced nephrotoxic model of AKI, cisplatin enhanced AEA but not 2-AG levels in renal tissue.^35^

To date, the mechanisms and conditions under which CB receptors are activated by ECs in the kidney—and subsequently the signaling cascades that result from this activation—have not been fully described. Studies have demonstrated conflicting results describing the role of AEA and CB_1_ receptor activation in mediating glomerular podocyte injury. Jourdan et al.^74^ showed that chronic exposure of human cultured podocytes to high glucose resulted in a significant upregulation in CB_1_ receptor gene expression, which is also associated with an increase in cellular AEA and 2-AG. This is associated with signs of inflammation and podocyte injury, which manifest as decreased podocin and nephrin and increased desmin gene expression.^74^ In contrast, Li et al.^75^ reported the protective functions of AEA following l-homocysteine (Hcys)-induced podocyte injury. AEA blocked Hcys-induced NLRP3 inflammasome activation in cultured podocytes and ameliorated podocyte dysfunction, ultimately precluding glomerular damage.^75^ Therefore, while the former study demonstrated that an increase in CB_1_ receptor gene expression accompanied by an upregulation in AEA and 2-AG is associated with podocyte injury, the latter study suggests that AEA exerts protective and anti-inflammatory effects in podocytes. Future studies are needed to investigate the role of EC ligands in CB receptor activation under varied conditions in renal health and disease.

## Conclusion

The EC system has been found to regulate a variety of functions in renal health and disease states. Various components of the EC system, namely the CB_1_ and CB_2_ receptors and their major physiologic activators (AEA and 2-AG), have been localized to a variety of renal cell subtypes across different species. Consequently, the activation or inhibition of CB_1_ and CB_2_ can significantly impact renal function with beneficial or adverse effects. Altered CB receptor expression has been demonstrated in a number of renal diseases, including nephropathy, CKD, and AKI. These findings have led to the investigation of CB receptor manipulation using pharmacological agents, which have partly pointed to the CB receptors as potential therapeutic targets for renal dysfunction. An important result of these studies was the demonstration that CB_1_ and CB_2_ receptors act via separate pathways and modulate distinct downstream targets in the kidney, despite a largely homogenous distribution in the renal system.

More recently, the EC system has been studied for its association with a variety of renal disease states. Collectively, these studies suggest that the activity of ECs should be examined separately from their interactions with CB receptors, as conflicting results were seen in the biological responses elicited by ECs and the activation of their receptors.

In summary, significant focus has been placed on evaluating the role of CB receptors in renal function, homeostasis, and pathophysiology. While these endeavors have contributed significantly to our understanding of the role of the EC system in the kidney, important areas of opportunity remain for future research, especially the role of EC ligands as mediators of EC system activity. At present, their role in renal physiology and pathophysiology remains to be fully elucidated. Furthermore, the clinical implications and relevance of EC system alteration will need to be further evaluated.^76^ Thus, while current data suggest that modulating EC system function and activity may provide a viable therapeutic intervention for renal dysfunction, future studies are essential to further elucidate the mechanisms through which ECs and CB receptors participate in renal physiology and disease, as well as the clinical context in which their stimulation or suppression could lead to beneficial or deleterious effects in the kidney.

## Abbreviations Used

2-AG: 2-arachidonoyl-*sn*-glycerol
AEA: anandamide
AKI: acute kidney injury
BUN: blood urea nitrogen
CB_1_: cannabinoid subtype-1
CB_2_: cannabinoid subtype-2
CKD: chronic kidney disease
CLP: cecal ligation and puncture
EC: endocannabinoid
FAAH: fatty acid amide hydrolase
GFR: glomerular filteration rate
Hcys: l-homocysteine
HTN: hypertension
MGL: monoacylglycerol lipase
ROS: reactive oxygen species
SA-AKI: sepsis-associated kidney injury
SHR: spontaneously hypertensive rat
STZ: streptozotocin
TAL: thick ascending limbs
UUO: unilateral ureteral obstruction
VEGF: vascular endothelial growth factor
ZDF: Zucker diabetic fatty
